# Retrospective log‐based quality assurance of real‐time MLC adaptation on a helical tomotherapy platform

**DOI:** 10.1002/acm2.70855

**Published:** 2026-10-08

**Authors:** Scott B. Crowe, Tanya Kairn

**Affiliations:** ^1^ Cancer Care Services Royal Brisbane and Women's Hospital Brisbane Australia; ^2^ School of Electrical Engineering and Computer Science University of Queensland Brisbane Australia; ^3^ School of Chemistry and Physics Queensland University of Technology Brisbane Australia

**Keywords:** adaptive radiation therapy, helical tomotherapy, quality assurance, respiratory motion management

## Abstract

**Background:**

Real‐time multileaf collimator (MLC) adaptation is available on some helical tomotherapy treatment units. However, these systems are not supplied with software that facilitates the retrospective review of adapted delivery data.

**Purpose:**

This study used in‐house software to complete a retrospective review of 1,566 treatment fractions with the aim of verifying the suitability and accuracy of MLC adaptations provided by a real‐time target tracking system.

**Methods:**

A data extractor tool was used to access files containing planned MLC sinogram, the telemetry treatment MLC sinogram, the telemetry delivery timing data, and the 3‐dimensional target offset data from each treatment fraction. These data were used to calculate target offsets in the beam's eye view so that expected adaptations could be evaluated and compared to the adaptations recorded by the MLC telemetry system. Further investigations were undertaken using dosimeter array measurements with a motion platform.

**Results:**

While MLC telemetry matched expected adaptations for the vast majority of treatment fractions, erroneous MLC leaf motions lasting >1 s were identified in four treatment fractions distributed across one lung and two prostate patients (amounting to 0.3% of the total number of fractions examined), producing dose discrepancies of up to 0.3 Gy which equated to 0.6% of the total prescription dose from the affected lung treatment. Phantom measurements demonstrated that a patch provided by the vendor successfully reduced the dosimetric impact of these errors to a negligible level.

**Conclusions:**

A retrospective review of 1,566 treatment fractions led to the identification of a rare error which was communicated to and subsequently patched worldwide by the vendor, exemplifying the importance of post‐treatment quality assurance of adaptive radiotherapy.

## 1 INTRODUCTION

Patient motion during radiotherapy beam delivery, or intrafraction motion, must be accounted for if planned target coverage is to be achieved. One method for managing motion is real‐time adaptation, also known as multi‐leaf collimator (MLC) tracking and motion synchronization.^1^, ^2^ This approach involves monitoring the position of the target (or surrogate fiducials) during treatment and adjusting the incident beam to account for the motion.

Real‐time adaptation is implemented on the Radixact helical tomotherapy treatment unit (Accuray, Madison, USA) with the Radixact Synchrony system.^3^, ^4^ During Radixact Synchrony treatments, the tomotherapy unit's dynamic jaws and binary MLCs are adjusted in response to changes in the longitudinal (IEC‐Y, superior‐inferior) and lateral (IEC‐X and IEC‐Z, left‐right and anterior‐posterior) position of the target in the beams eye view. The target position is verified using planar x‐ray images acquired during treatment, by identifying either the (markerless) tumor within the lung or implanted markers. For lung and abdominal treatments, where the dominant motion is regular respiratory motion, surrogate light‐emitting diodes (LEDs) placed on the patient are monitored and correlated with target motion.

The verification of accurate real‐time adaptation is critical, particularly as it is generally accompanied by a reduction in treatment margins (for example, the internal target volume, ITV). The verification of real‐time adaptation and Radixact Synchrony specifically is recommended by the AAPM TG‐264 and TG‐306 reports.^2^, ^3^ Various validation studies have been performed and published as part of Radixact Synchrony system commissioning,^4^, ^5^, ^6^ benchmarking,^7^, ^8^, ^9^, ^10^, ^11^, ^12^, ^13^ and pre‐treatment patient‐specific quality assurance (QA) activities.^4^, ^5^, ^14^

There is a need for an alternative to measurement‐ based approaches, to allow validation of patient treatment fraction deliveries, especially in situations where real‐time adaptation during treatment makes pre‐treatment or retrospective quality assurance measurements challenging. One alternative is the analysis of log (or telemetry) files, which contain beam, gantry, collimator and couch data from treatment fraction deliveries that can be compared against planned parameters.^15^

Log files have been used to characterize the performance of motion management techniques such as gating,^16^ and used to validate non‐Synchrony Radixact deliveries.^17^, ^18^ The AAPM TG‐264 report specifically recommends that log files describing treatment delivery be recorded by the treatment system for use in post‐treatment quality assurance (QA) and in the active monitoring of system performance.^19^ The analysis of these treatment log file data was an action identified by a prospective hazard analysis of the Radixact Synchrony system.^20^

For non‐Synchrony Radixact treatment deliveries, the MLC behavior during patient treatment fraction deliveries can be evaluated using the Accuray Delivery Analysis software.^17^ This software allows comparison between planned and telemetry MLC leaf open times (derived from MLC optical position sensors) and exit detector fluence measurements. The Delivery Analysis software also performs a dose calculation using the telemetry MLC data, to evaluate the potential impact of any deviation.

However, for Radixact Synchrony treatments, telemetry and exit detector MLC leaf open times are not shown in Delivery Analysis, and the only treatment fraction delivery data available are Synchrony x‐ray radiographs, target offset and Synchrony tracking metrics. To overcome this limitation in Delivery Analysis, and to facilitate batch analysis, in‐house Python code has been developed,^21^, ^22^ and has been used to evaluate the dosimetric benefit of real‐time adaptation.^23^

This study provides an overview of one implementation of an in‐house software package^21^, ^22^ for the analysis and verification of real‐time MLC adaptation treatment log data generated by the Accuray Radixact Synchrony system, the identification of erroneous leaf behavior, the validation of a vendor‐issued bug fix, and includes illustrative examples of accurate and erroneous real‐time MLC adaptation.

## 2 METHODS

### 2.1 Data collection and analysis

Data generated and recorded during Radixact treatment planning and fraction deliveries, including treatment plans, exit detector measurements, and log or telemetry data were retrospectively collected for patient and phantom using the Accuray Patient Data Extractor (PDE) tool. These data have been routinely collected for all Synchrony deliveries on the two Radixact systems in one radiotherapy department.

The files exported using the PDE tool were parsed using the in‐house Python code.^21^, ^22^ For this study, the relevant data included the planned MLC sinogram, the telemetry treatment MLC sinogram, the telemetry delivery timing data, and the 3‐dimensional target offset data. Each sinogram is a 2D array of values defining the fractional leaf open time of each MLC in each treatment projection. Treatment projections are analogous to treatment control points and represent 7.06° of gantry rotation, typically delivered over 0.2–0.6 s. The telemetry sinogram and motion data have timestamps, allowing the target position, planned MLC openings and gantry rotations to be registered.

For each projection the IEC‐X and IEC‐Z target position and gantry position were used to calculate the target offset from the planned position in the beam's eye view. This value was compared against a threshold leaf adaptation value of 4 mm. The 4 mm threshold value was iteratively optimized to maximize agreement with observed adaptations, is consistent with vendor advice (slightly larger than half the MLC leaf thickness of 6.25 mm)^24^, and is comparable to the estimated 4.3 mm threshold experimentally determined by Miura et al.^25^

Where the difference between the current leaf adaptation and beam's eye view target offset exceeded the threshold, an “expected adaptation” was calculated by shifting the leaf open time values within each projection. The “telemetry leaf adaptation” was calculated as the shift between the planned and telemetry sinogram projections.

Planned and telemetry leaf open time values are recorded on a projection scale, that is, they are updated with each 0.2 ‐ 0.6 s projection. However, the underlying Synchrony target offset calculations and the delivery of MLC position instructions occurs more frequently, every 0.01 s.^24^ To reduce potential disagreement between expected and telemetry sinograms due to sub‐projection adaptations, the target offset and expected adaptation were calculated 20 times per projection, with leaf open time calculated across each sub‐projection (by up‐sampling and down‐sampling the sinogram data before and after adaptation calculations).

For each projection, the total planned and telemetry fractional leaf open time was also calculated, and the difference between the two values compared against a threshold of 0.5, to allow identification of erroneous fluence variations, or substantial variation in the number of open leaves. These variations were categorized as erroneous, as Synchrony adapts to motion by shifting the jaw and MLC defined field while maintaining field dimensions. The frequency and duration of these variations between planned and telemetry sinograms were recorded across prostate and lung patient cohorts.

The total fractional leaf open time threshold of 0.5 was selected with consideration of expected, non‐erroneous variations in leaf open times. Variations in leaf latency, as measured during daily QA, are automatically compensated for on the Radixact platform by adjustment of leaf open times during treatment delivery.^26^ A lower threshold (close to 0) could incorrectly characterize leaf timing corrections as erroneous variations. Similarly, a higher threshold (close to 1) could incorrectly fail to identify an erroneous fluence variation as having occurred due to leaf timing corrections. Leaf timing corrections can be up to 5% of total fractional leaf open times (as seen in results of this study, where total fractional leaf open times were between 0 and 10).

The matplotlib library was then used to generate figures illustrating leaf adaptations, including the planned sinogram, target offset, expected and telemetry leaf adaptation, and total planned and telemetry leaf open time across each projection, with expected and erroneous variations highlighted.

The code for parsing plan and telemetry data, calculating the target offset in beam's eye view, estimating expected adaptation, and plotting the results shown in this paper can be found in the radixact modules of the pyradlib code repository,^22^ with snippets provided as Supporting Materials 1.

### 2.2 MLC adaptation to patient motion

For this study, MLC leaf adaptation was examined for 1,566 Radixact Synchrony treatment fractions, including 1,533 prostate treatment fractions and 33 lung tumor treatment fractions. The prostate treatments used conventional fractionation (2‐3 Gy per fraction) and jaw separations of 2.5 cm for all treatments, using Synchrony fiducial tracking. The lung treatments included a mix of conventional and stereotactic body radiotherapy (SBRT) fractionations, and 1.0 and 2.5 cm jaw separations, using markerless Synchrony target tracking and respiratory motion modelling. The figures generated with the in‐house software were evaluated for any discrepancy between expected and telemetry leaf adaptation sinograms, as a quality assurance activity.

Analysis of the patient treatment fractions resulted in the identification of erroneous leaf behavior, presenting as a leaf remaining incorrectly open or closed for multiple gantry angle projections. This issue was reported to Accuray for investigation.

Dose estimates for the impact of the erroneous leaf behavior were made based on simple rotational projections, shown in Supporting Materials 2. The simple estimate involved the multiplication of the fractional treatment dose (e.g., 12 Gy), by the pitch (e.g., 0.125) to account for output for a given gantry rotation, and a factor describing the proportion of a gantry that any given tissue might spend in the beamlet corresponding to an erroneously open leaf.

### 2.3 MLC adaptation to phantom motion

To further investigate the potential dosimetric impact from the MLC leaf error identified using the method in section 2.2, phantom measurements were performed. A Radixact treatment plan using Synchrony respiratory tracking was optimized to deliver 2 Gy per fraction to a 1 cm diameter target using a 1 cm jaw and pitch of 0.5. The delivery was planned for and measured with a PTW Octavius 1000 SRS detector array (PTW, Freiburg, Germany) in an Octavius II phantom with custom inhomogeneity insert, placed on a CIRS 1D dynamic platform (Sun Nuclear Corporation, Melbourne, USA) rotated 30° to achieve both IEC‐X and IEC‐Y motion.^14^ The motion platform was programmed with a ±10 mm cos^4^ amplitude and 4 s cycle time waveform, translating to approximately 8 mm IEC‐Y and 5 mm IEC‐X motion.

The use of respiratory tracking and respiratory motion was expected to be more likely to trigger an MLC leaf error (based on observations of patient treatment errors). The use of a low dose, short treatment length and high pitch delivery was expected to produce a larger dose error as a fraction of the delivered dose, with a reduced treatment fraction delivery time, allowing more efficient error detection. The basic waveform used was described by Sano et al.^27^ in a study of Synchrony tracking performance, and the 5 mm IEC‐X motion would ensure MLC adaptation occurred during delivery. The relatively simple waveform also allowed visual verification that target tracking was occurring as expected during and after treatment fraction delivery.

The Octavius array measurements were exported as DICOM dose planes using Verisoft (version 8.1, PTW Freiburg, Germany), and compared using pymedphys with gamma criteria of 2% of global dose maximum, 2 mm distance‐to‐agreement (as used for conventionally fractionated treatments in the department), and 3% of global dose maximum, 1 mm distance‐to‐agreement (as used for SBRT treatments in the department), with a low‐dose threshold of 5%.^28^

Dose estimates for the impact of the erroneous leaf behavior were again made based on simple rotational projection, and are shown in Supporting Materials 2.

### 2.4 Investigation of vendor response to MLC leaf error

The phantom measurements described in section 2.3 were repeated following a vendor‐initiated software update, to confirm that the MLC errors identified through the work described in section 2.2 were no longer able to significantly affect treatment delivery. Specifically, additional phantom measurements were designed and completed in order to validate the correct behavior of the system interrupt (described in section 3.2), and to measure any residual dose error. The basic waveform used previously was modified to increase the frequency of the MLC error, with a reduced cycle time (2.6 s instead of 4 s) and a 10 mm offset in the center of motion (5 mm in IEC‐X). These changes would increase the proportion of the treatment fraction delivery that the target offset was close to the threshold for adaptation, which Accuray had suggested was the cause of the error.

## 3 RESULTS

### 3.1 MLC adaptation to patient motion

For all fractions, there were small variations between the expected and telemetry leaf open times. These small variations were not necessarily related to Synchrony, with potential causes including leaf timing corrections for MLC latency (discussed in section 2.1), handling of projections with very short leaf‐open times, and optical sensor temporal resolution. These variations were typically <5% of leaf‐open time within a projection (or within <0.03 s).

Similarly, there were small disagreements in the number of projections where adaptation was expected and where it was observed in telemetry data, typically <5%, due to differences between the retrospective modelling of adaptation from telemetry data and the actual Synchrony implementation. Uncertainties include the estimated 4.0 mm threshold (the actual value is not published), the temporal resolution of telemetry data (>0.1 s, whereas Synchrony performs target offset calculations and provides MLC position instructions every 0.01s), and the model not accounting for MLC latency (Synchrony estimates target offset at time leaf will have moved to position).

Figure 1 shows an example of successful motion adaptation for a prostate Synchrony treatment, with (A) the planned sinogram, (B) the detected motion of the treatment target, (C) the result of predicting whether or not MLC adaptation was expected, (D) an indication of when adaptation was predicted in relation to the planned sinogram, (E) an indication of when adaptation occurred in relation to the planned sinogram and (F) an illustration of agreement between the planned and delivered leaf‐open times, all plotted against the sinogram projection number for this treatment fraction. In this example, there is an approximately 10 mm IEC‐Z target offset for the duration of treatment, resulting in adaptation occurring near gantry angles 90° and 270°.

**FIGURE 1:**
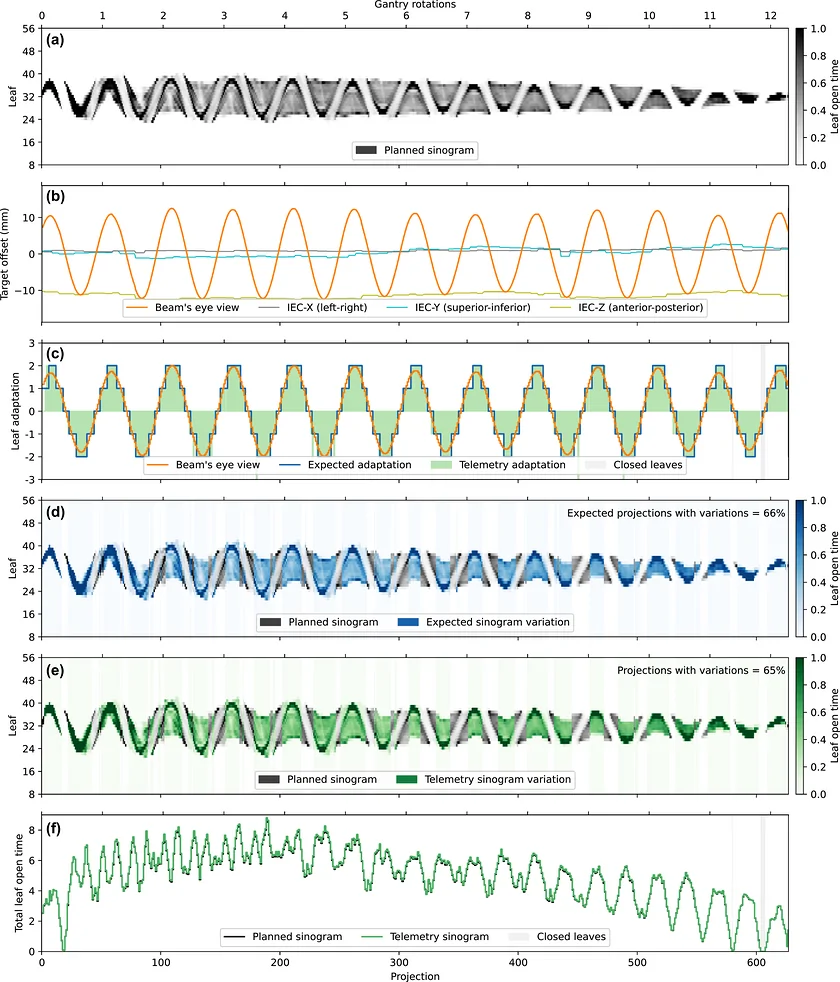
Accurate adaptation of prostate fiducial target tracking Synchrony treatment fraction. a: the planned sinogram for this treatment. b: information used to predict the expected treatment adaptation, where the detected motions of the treatment target in the IEC‐X, IEC‐Y and IEC‐Z directions are respectively shown as grey, blue and brown lines, and the vector sum of IEC‐X and IEC‐Z motion in the beam's‐eye‐view is shown as a thick orange line. c: the beam's‐eye‐view target motion (thick orange line) is shown overlying the expected adaptation calculated by the in‐house software (blue line) with green shading indicating when MLC adaptation occurred and grey shading showing when adaptation was not needed because all MLCs were closed. d: blue shading over the planned sinogram shows when adaptation was expected. e: green shading over the planned sinogram shows when adaptation occurred. f: illustration of agreement between the leaf‐open times from the planned (black) and telemetry (green) sinograms for each projection, indicating that the number of open MLCs during each projection was consistent.

For 23 of the 1,566 Synchrony treatment fractions evaluated (1.1%), an erroneous sinogram variation was observed. For 13 of these treatment fractions, the error was transient and had a duration of less than 0.5 seconds (or 1 projection). For 4 treatment fractions (0.3%) the error had a duration greater than 1 second (or at least 5 projections), which was interpreted as an unambiguous error rather than a transient variation. The 4 treatment fractions with erroneous variations were reported to Accuray. Table 1 summarizes these sinogram variations for each cohort.

**TABLE 1:** Summary of treatment fractions with MLC adaptation for erroneous sinogram variations for the lung and prostate cohorts

			Number (and percentage) of fractions with erroneous variations				
Cohort	Number (and percentage) of fractions with MLC adaptation	Number (and percentage) of fractions with no erroneous variations	Duration ≤0.5 s	Duration 0.5–1 s	Duration >1 s	Maximum erroneous projections duration(s)	Maximum dose discrepancy (and percentage of total prescribed dose)
Lung	30 (91%)	30 (91%)	1 (3%)	1 (3%)	1 (3%)	16.7	≤0.3 Gy (≤0.6%)
Prostate	892 (58%)	1513 (99%)	12 (0.8%)	5 (0.3%)	3 (0.2%)	18.5	≤0.15 Gy (≤0.3%)
All treatments (lung + prostate)	922 (59%)	1543 (99%)	13 (0.8%)	6 (0.4%)	4 (0.3%)	18.5	≤0.3 Gy (≤0.6%)

The longest duration erroneous variations have been shown in Figure 2 and Figure 3. Full versions of these figures including target offset and examples for other treatment fraction deliveries have been included in Supporting Materials 3.

**FIGURE 2:**
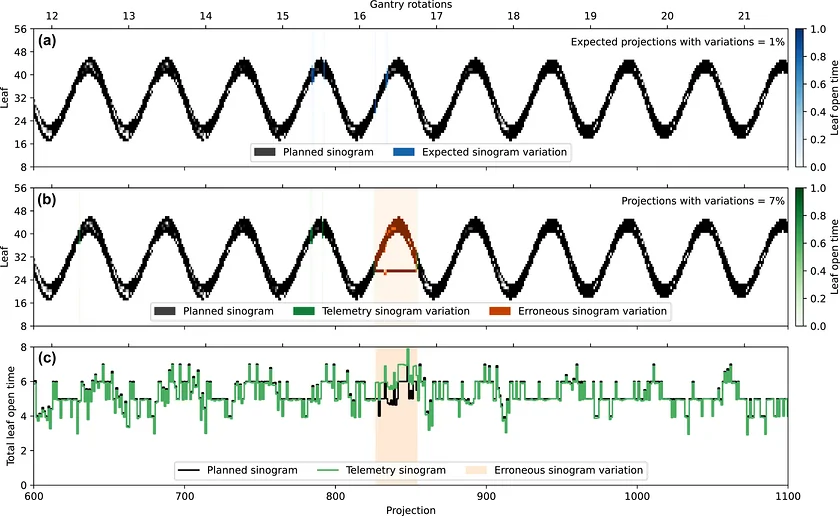
Erroneous leaf positioning during lung respiratory target tracking Synchrony treatment fraction with minimal MLC adaptation. a: the planned sinogram for this treatment overlaid with the sinogram that was expected based on detected target motion (green). b: the planned sinogram overlaid with the delivered sinogram recorded by the telemetry system (green) with a period of erroneous adaptation highlighted in orange. c: illustration of disagreement (highlighted in orange) between the leaf‐open times from the planned (black) and telemetry (green) leaf‐open times for each projection, indicating that an erroneous sinogram variation lasting 16.7 s occurred, starting from projection 827.

**FIGURE 3:**
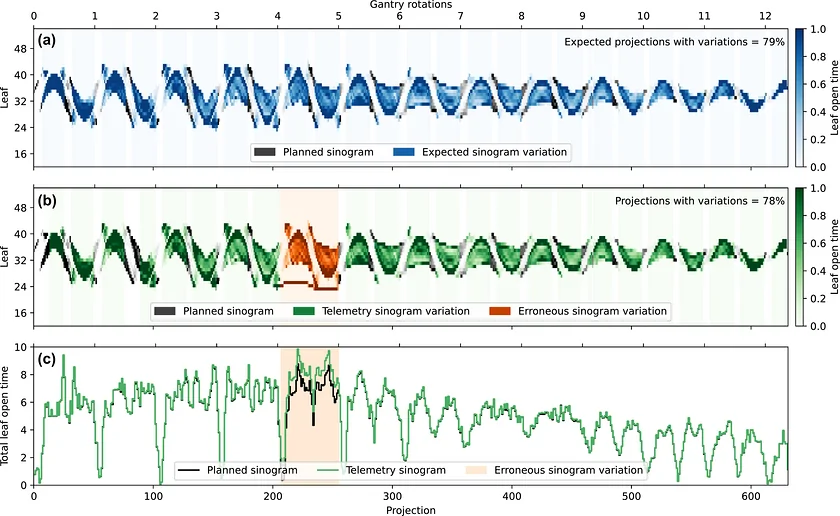
Erroneous adaptation during a prostate fiducial tracking Synchrony treatment fraction with frequent MLC adaptations. a, b and c are as defined for Figure 2, except that the erroneous sinogram variation lasting 18.5 s occurred, starting from projection 206.

Figure 2 shows an instance of erroneous variation during a lung fraction. In this instance, minimal IEC‐X and IEC‐Z target motion produced minimal MLC adaptation during the projections shown. Following one MLC adaptation event, a relatively central leaf has erroneously remained open for 16.7 s, or approximately half a gantry rotation.

Figure 3 presents another example of erroneous leaf opening for a prostate treatment fraction. Unlike the IEC‐X target offset in the example in Figure 1, in this example target offset in both IEC‐X and IEC‐Z dimensions has resulted in near constant leaf adaptation.

The maximum dosimetric impact of the erroneously open leaves was estimated to be 0.3 Gy, as shown in Supporting Materials 2. The dose difference appears as a ring centered on the treatment isocentre, with a radius and magnitude depending on the offset of the affected leaf from the central axis of the beam. For example, for leaf 28 in the lung SBRT example, the ring is located approximately 25 mm from the isocentre (an offset of 4 leaves with thickness 6.25 mm each), and the magnitude is 0.2 multiplied by the fractional dose and pitch.

### 3.2 Vendor response to MLC leaf error

Accuray performed an internal investigation of the MLC leaf error that was identified using the method described in section 2.2 and identified a bug in the method used by the system to prevent unnecessary (very brief, e.g., 50 ms) adaptations. This bug would occur when the target offset was close to the threshold for adaptation.

Accuray distributed a customer notification to all Radixact Synchrony users in February 2026 reporting on the issue identified in this study.^29^ The issue was addressed in an update to the Radixact Treatment Delivery System software (version 3.0.3.2) made available to Radixact users worldwide in mid‐2026. In this updated version, a system interrupt is triggered when a deviation between the planned and adapted number of open leaves occurs continuously for 500 ms, or for at least 600 ms in a 700 ms period.

### 3.3 MLC adaptation to phantom motion before and after bug fix

Erroneous leaf behavior was observed during a measurement of a phantom treatment delivery, as shown in Figure 4, lasting approximately half a gantry rotation and consisting of a leaf remaining erroneously closed instead of opening in response to adaptation. Similarly erroneous leaf behavior successfully triggering a beam interrupt after the implementation of the Accuray bug fix is shown in Figure 5.

**FIGURE 4:**
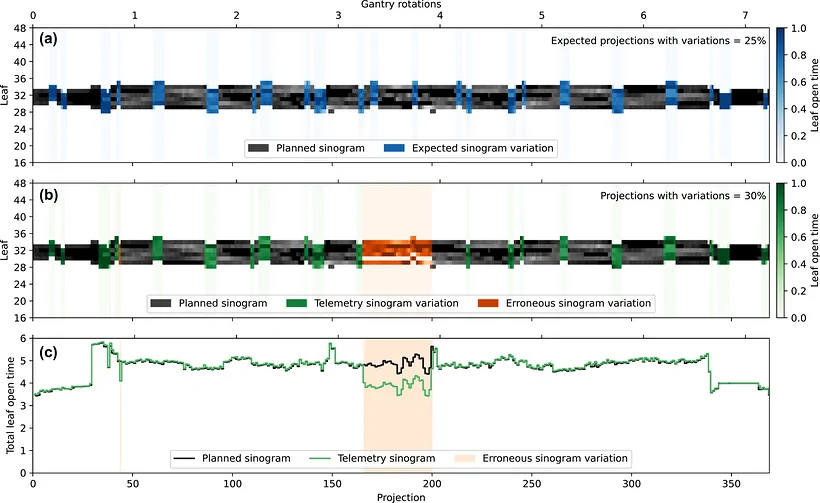
Erroneous adaptation during a lung respiratory target tracking Synchrony phantom treatment fraction. a, b and c are as defined for Figure 2, except that the erroneous sinogram variation started from approximately projection 179.

**FIGURE 5:**
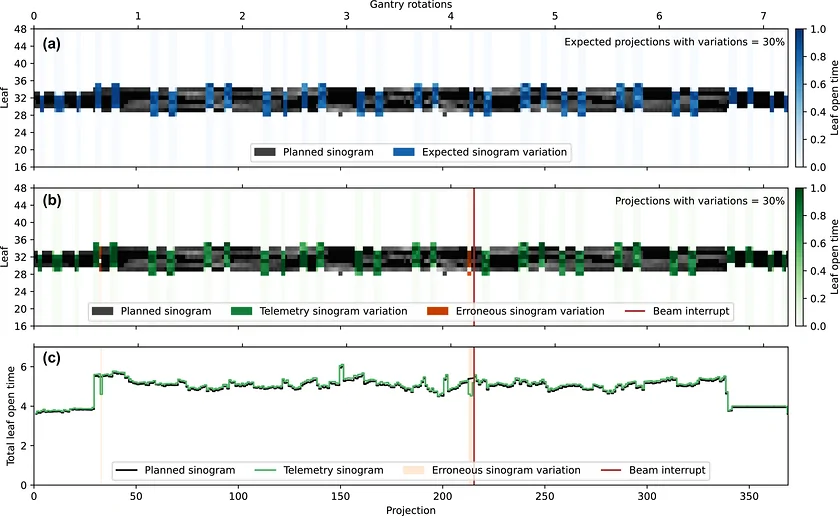
Erroneous adaptation during a lung respiratory target tracking Synchrony phantom treatment delivery, triggering a system interruption to prevent dosimetric error. a, b and c are as defined for Figure 2, except that the erroneous sinogram variation occurred for two projections, starting at approximately projection 220. This error immediately caused a system interrupt (or interlock, indicated by dark orange vertical line), preventing a dosimetric error.

Measurements for the static and Synchrony deliveries are compared in Figure 6, before the bug fix, and in Figure 7, after the bug fix. Figure 6 shows a 15% dose depletion arising from the erroneously closed MLC, which equates to a dose difference of approximately 0.3 Gy in this 2 Gy treatment fraction. The corresponding dose estimate for this instance of the leaf error was between 0.26 and 0.32 Gy (as leaves 30 and 31 were erroneously closed during the error), shown in Supporting Materials 2. This provides broad confirmation of the dose discrepancy estimated for the patient treatments in Section 3.1.

**FIGURE 6:**
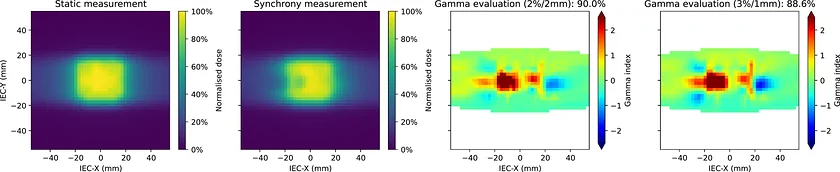
Dosimetric comparison of static and Synchrony measurements with erroneous leaf behavior prior to system interrupt fix, as measured with an Octavius 1000 SRS detector.

**FIGURE 7:**
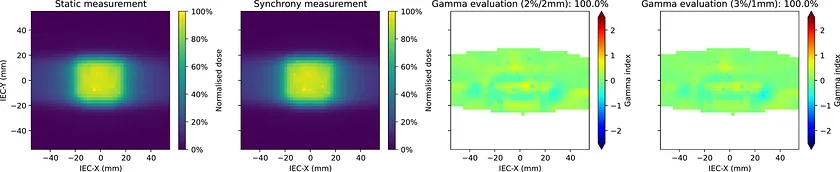
Dosimetric comparison of static and Synchrony measurements with erroneous leaf behavior and system interrupt fix, as measured with an Octavius 1000 SRS detector.

The dose depletion due to the leaf error shown in Figure 4 resulted in gamma pass rates ≤90% between static and Synchrony deliveries in Figure 6. The corresponding gamma pass rates are 100% for the interrupted delivery in Figure 7, providing confidence in the bug fix supplied by Accuray.

## 4 Discussion

In‐house software was developed which allowed erroneous MLC leaf behavior lasting more than 1 s to be detected in a small percentage (0.3%) of the 1,566 Radixact Synchrony treatment fractions evaluated in this study. It was expected that the duration of the error would typically be less than one gantry rotation, due to the “sweeping” of open leaves for lateral targets, and the change in beams‐eye profile of larger rounded targets that might be treated with central leaves.

Errors not lasting more than one gantry rotation might result in dose deviations of about 0.3 Gy. While this is a relatively high percentage of the fractional dose (10% of 3 Gy prostate treatments and 15% of 2 Gy phantom treatments), it would be relatively small as a percentage of total prescriptions (0.5%). The estimates of dose error in this study were consistent with Accuray‐provided dose calculations performed using their proprietary internal dose calculation algorithm CalcZilla.

There was a possibility that a leaf error could occur at the last programmed leaf closure for a given leaf, which would cause the leaf to remain open for the remainder of the treatment. If a leaf were to remain open for multiple gantry rotations, the impact could have been non‐negligible, e.g., the fractional prescribed dose for a lung SBRT (12 Gy) multiplied by the leaf projection factor (0.2), would be 2.4 Gy, or 5% of prescription. There is insufficient data to provide an accurate estimate of the likelihood of this occurring. However, if the projection on which the error occurs could be assumed to be random, the probability of the error occurring during a projection with the left‐most or right‐most open leaf of a gantry rotation would conservatively be 4% of instances of leaf errors within the gantry rotation. That is, the likelihood of this occurring for a given fraction would be 0.012%.

The frequency of the erroneous leaf behavior may vary with the MLC sinogram and target motion, which would vary with treatment site. The 0.3% incidence rate reported in this study was derived from a dataset where 98% of analyzed fractions were from prostate treatments, and the 0.3% value may not be representative for a department, for example, that primarily uses Synchrony for lung treatments, where target motion is periodic and MLC sinograms can be sinusoidal in appearance.

Based on the results shown in Table 1, the system interrupt implemented by Accuray (which triggers with a leaf variation of 0.5 s duration) may occur in approximately 0.6% of treatment fractions. In addition to implementing a fix to the leaf issue, Accuray has now developed software for Radixact Synchrony customers with similar functionality to the software used in this study: the Radixact MLC data viewer and Radixact Synchrony Leaf Analyzer; allowing analysis of log file data for specific patients.

For conventional C‐arm linacs, data from portal images can be used to validate the accuracy of treatment delivery as well as the accuracy of treatment data recorded in log files.^30^, ^31^, ^32^ For Radixact tomotherapy treatment units, it should be possible to use sinogram data from the exit detector to provide an analogous verification of treatment delivery and log file accuracy. For Synchrony treatments however, it is not possible to use information from the exit detector directly because the fluence at the detector location is affected by the longitudinal motion adaptation performed by the Radixact jaws. When the Synchrony system detects that the treatment target is displaced from its planned position in the longitudinal (IEC‐Y) direction, the jaws move to adapt to match that displacement, reducing the signal to the exit detector until the target and the jaws move back to their central position. This effect is particularly obvious for treatments planned with a 1 cm jaw aperture. Use of the detector sinogram data to verify Synchrony treatment delivery would require modelling of this effect, in combination with the variable attenuation of the treatment beam by the patient anatomy. There is further potential for these datasets to be used for back‐projected dose calculations.

## 5 CONCLUSION

The in‐house software developed for this study allowed a retrospective review of 1,566 Radixact Synchrony treatment deliveries to be completed, leading to the identification of a rare error which was communicated to and subsequently patched worldwide by the vendor. The results of this study exemplify the importance of post‐treatment quality assurance of adaptive radiotherapy by highlighting some rare cases where issues with the handling of MLC adaptation have been observed.

## AUTHOR CONTRIBUTIONS

**Scott B. Crowe**: conceptualization; formal analysis; investigation; methodology; project administration; software; validation; visualization; writing–original draft. **Tanya Kairn**: investigation; methodology; validation; writing–original draft; writing–review and editing.

## CONFLICT OF INTEREST STATEMENT

The authors have no financial or non‐financial interest in any vendor mentioned in the paper. The affiliated Royal Brisbane and Women's Hospital have a product evaluation agreement with Accuray and provide customer feedback as a member of the Accuray Adaptive Consortium.

## FUNDING INFORMATION

The authors have nothing to report.

## ETHICAL APPROVAL

The study was approved as a quality evaluation activity by the Metro North Health Human Research Ethics Committee A (EX/2023/MNHA/97008).

## Supporting information




**Supporting File**: acm270855‐sup‐0001‐SuppMat.pdf.

**Supporting File**: acm270855‐sup‐0002‐SuppMat1.pdf.

**Supporting File**: acm270855‐sup‐0003‐SuppMat2.pdf.

## Data Availability

Data that support the findings of this study are available in the supporting material and from the corresponding author, Scott B. Crowe, upon reasonable request.
