# Supplementary material for: Retrospective log‐based quality assurance of real‐time MLC adaptation on a helical tomotherapy platform
Source: J Appl Clin Med Phys. 2026 Oct 8;27(10):e70855. doi: 10.1002/acm2.70855 (PMC13650519; doi:10.1002/acm2.70855)
Supplement: Supplementary file 1 [file ACM2-27-e70855-s001.pdf]

Dose estimation worksheet

This Python worksheet illustrates how the dosimetric impact of an erroneously open leaf can be estimated. Two results are shown:

1. Dose difference map for open leaf 28, for a lung SBRT fraction with a 12 Gy fractional dose and pitch of 0.125.
2. Table showing maximum intensity factor for various open leaves, to be multiplied against fractional dose and pitch.

In [21]:
```
# Import statements
import matplotlib.pyplot as plt
import numpy as np
import polars as pl
from skimage.draw import polygon
```

In [22]:
```
# Default parameters
source_iso_distance = 850
mlc_focus_iso_distance = 849.5
mlc_leaf_thickness_at_iso = 6.25
total_number_of_leaves = 64
projection_image_length = 2000
projection_image_bore_radius = 425
projection_angle_step = 0.1
```

In [23]:
```
# Helper function to calculate location of source in a fluence map for a given gantry angle.
def source_position(gantry_angle):
    return (
        (projection_image_length / 2) - np.cos(gantry_angle) * source_iso_distance,
        (projection_image_length / 2) + np.sin(gantry_angle) * source_iso_distance,
    )
```

In [24]:
```
# Function to calculate location of projection in fluence map.
def projection_position(gantry_angle):
    return source_position(gantry_angle + np.pi)
```

In [25]:
```
# Function to calculate simple ray projection for a given source position.
def offset_projection_positions(
    gantry_angle, inner_leaf_offset_at_iso, outer_leaf_offset_at_iso
):
    point1_x = (
        (projection_image_length / 2)
        - np.cos(gantry_angle + np.pi) * source_iso_distance
    ) + np.sin(gantry_angle) * (inner_leaf_offset_at_iso * 2)
    point1_y = (
        (projection_image_length / 2)
        + np.sin(gantry_angle + np.pi) * source_iso_distance
    ) + np.cos(gantry_angle) * (inner_leaf_offset_at_iso * 2)
    point2_x = (
        (projection_image_length / 2)
        - np.cos(gantry_angle + np.pi) * source_iso_distance
    ) + np.sin(gantry_angle) * (outer_leaf_offset_at_iso * 2)
    point2_y = (
        (projection_image_length / 2)
        + np.sin(gantry_angle + np.pi) * source_iso_distance
    ) + np.cos(gantry_angle) * (outer_leaf_offset_at_iso * 2)
    return source_position(gantry_angle), (point1_x, point1_y), (point2_x, point2_y)
```

In [26]:
```
# Function to produce a simple ray projection intensity map, for a full gantry rotation.
def projection_intensity_map(open_leaf_number):
    inner_leaf_offset_at_iso = (
        open_leaf_number - (total_number_of_leaves / 2)
    ) * mlc_leaf_thickness_at_iso
    if open_leaf_number <= (total_number_of_leaves / 2):
        outer_leaf_offset_at_iso = inner_leaf_offset_at_iso - mlc_leaf_thickness_at_iso
    else:
        outer_leaf_offset_at_iso = inner_leaf_offset_at_iso + mlc_leaf_thickness_at_iso
    projection_fluence = 1 / 360 * projection_angle_step
```

```python
    img = np.zeros(
        (projection_image_length, projection_image_length)
    )  # first num is height, second is width
    for gantry_rotation in np.arange(0, 360, projection_angle_step):
        source_pos = source_position(np.deg2rad(gantry_rotation))
        source_pos, point1, point2 = offset_projection_positions(
            np.deg2rad(gantry_rotation),
            inner_leaf_offset_at_iso,
            outer_leaf_offset_at_iso,
        )
        r = [int(source_pos[0] + 0.5), int(point1[0] + 0.5), int(point2[0] + 0.5)]
        c = [int(source_pos[1] + 0.5), int(point1[1] + 0.5), int(point2[1] + 0.5)]
        rr, cc = polygon(r, c)
        img[rr, cc] += projection_fluence
    # mask everything outside 425 mm - bore size
    xx, yy = np.mgrid[:projection_image_length, :projection_image_length]
    distance_squared = (xx - (projection_image_length / 2)) ** 2 + (
        yy - (projection_image_length / 2)
    ) ** 2
    img[distance_squared >= projection_image_bore_radius**2] = 0
    return img
```

In [27]:

```python
# Function to scale a ray projection intensity map to dose, for a full gantry rotation.
def dose_map(open_leaf_number, fraction_treatment_dose, pitch):
    return projection_intensity_map(open_leaf_number) * fraction_treatment_dose * pitch
```

In [28]:

```python
# Function to plot a dose map using matplotlib.
def plot_dose_map(open_leaf_number, fraction_treatment_dose, pitch):
    img = dose_map(open_leaf_number, fraction_treatment_dose, pitch)
    fig, axes = plt.subplots(1, 2, figsize=(12, 5))
    im1 = axes[0].imshow(img[500:1500, 500:1500], extent=[-500, 500, 500, -500])
    fig.colorbar(im1, ax=axes[0], label="Gy")
    axes[0].set_title("Projected dose estimate, \nfor full 1.5 Gy gantry rotation")
    axes[0].set_xlabel("IEC-X (mm)")
    axes[0].set_ylabel("IEC-Z (mm)")
    axes[1].plot(np.arange(-500, 500, 1), img[500:1500, 1000])
    axes[1].set_title(
        "Profile of projected dose estimate, \nfor full 1.5 Gy gantry rotation"
    )
    axes[1].set_xlabel("IEC-X (mm)")
    axes[1].set_ylabel("Dose (Gy)")
    fig.tight_layout()
    return fig
```

In [29]:

```python
# Plot dose map for lung SBRT case.
print("Dose estimate for lung SBRT case")
fig = plot_dose_map(open_leaf_number=28, fraction_treatment_dose=12, pitch=0.125)
```

```
Dose estimate for lung SBRT case
```

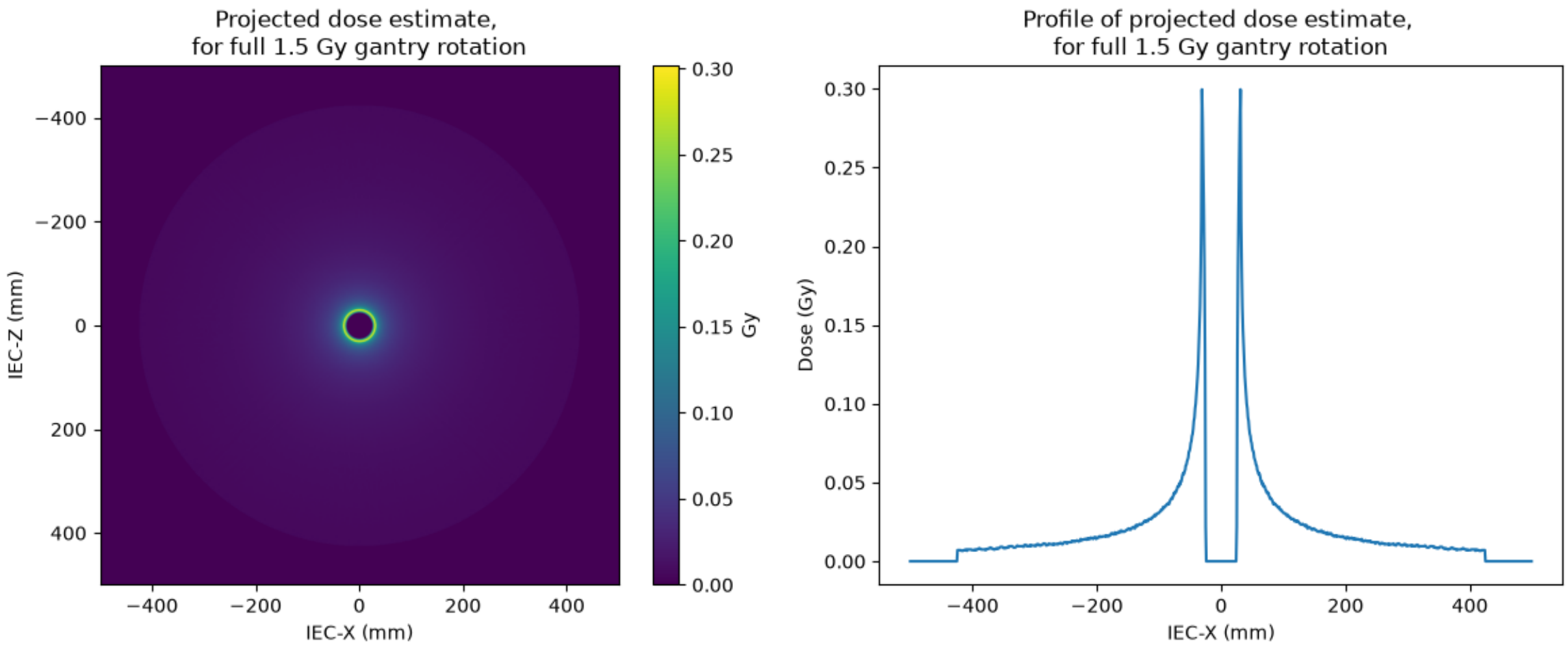

In [30]:

```python
# Estimate scaling factor for multiplication by dose and pitch for each possible leaf.
scaling_factor_dict = []
for leaf in np.arange(1, 33):
    scaling_factor_dict.append(
```

```
        {
            "leaf_number": leaf,
            "max_intensity_factor": np.max(projection_intensity_map(leaf)),
            "distance_from_isocentre": ((total_number_of_leaves/2)-leaf) * mlc_leaf_thickness_at_iso
        }
    )
pl.DataFrame(scaling_factor_dict)
```

Out[30]: shape: (32, 3)

leaf_number	max_intensity_factor	distance_from_isocentre
i64	f64	f64
1	0.076944	193.75
2	0.0775	187.5
3	0.079167	181.25
4	0.080833	175.0
5	0.0825	168.75
…	…	…
28	0.201111	25.0
29	0.226111	18.75
30	0.261944	12.5
31	0.327222	6.25
32	1.0	0.0
