# Supplementary material for: Retrospective log‐based quality assurance of real‐time MLC adaptation on a helical tomotherapy platform
Source: J Appl Clin Med Phys. 2026 Oct 8;27(10):e70855. doi: 10.1002/acm2.70855 (PMC13650519; doi:10.1002/acm2.70855)
Supplement: Supplementary file 2 [file ACM2-27-e70855-s002.pdf]

Supporting Materials 3 provides complete examples of retrospective QA results for various treatment fraction sinograms.

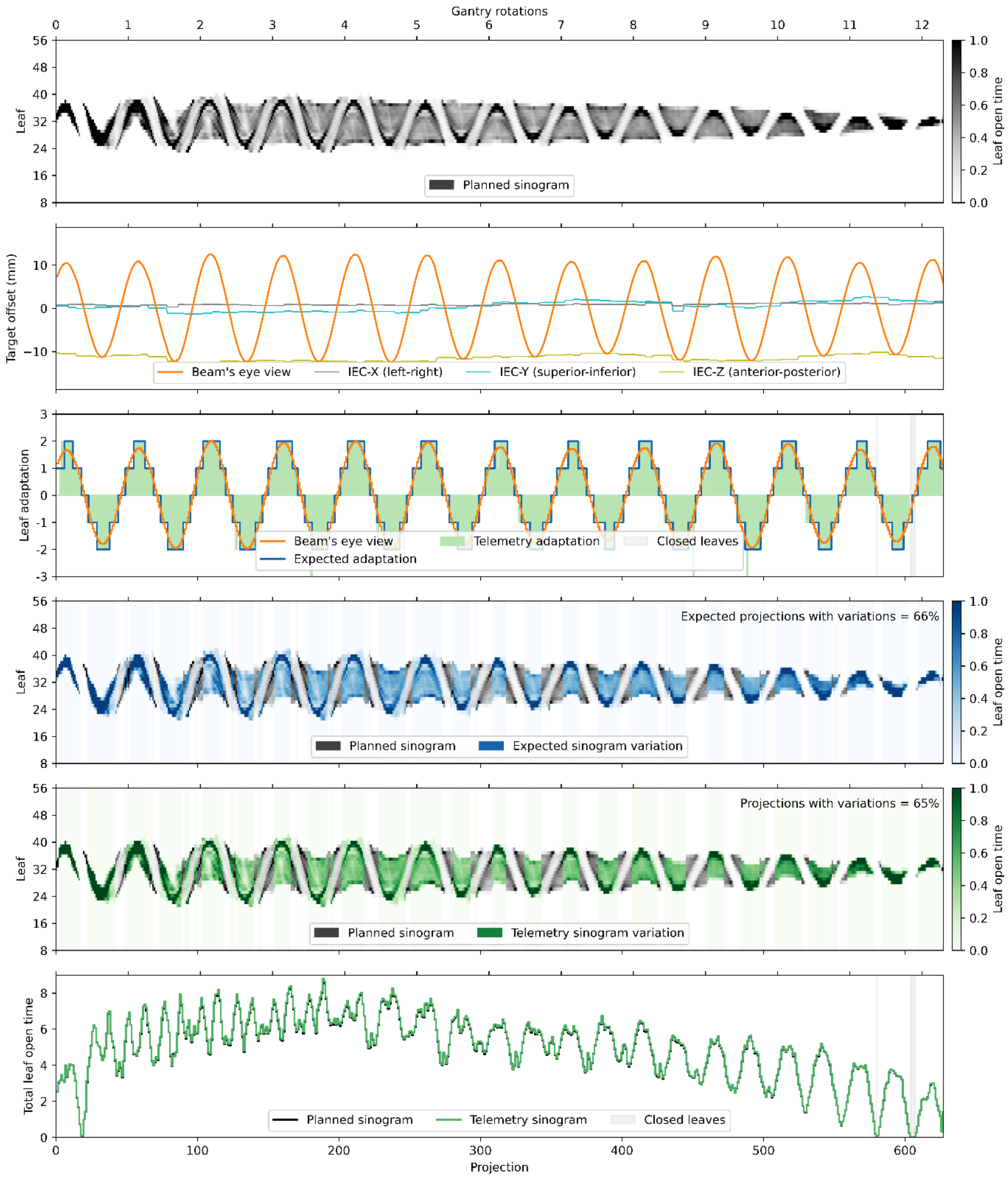

Figure 1 Example of accurate leaf behavior for prostate treatment

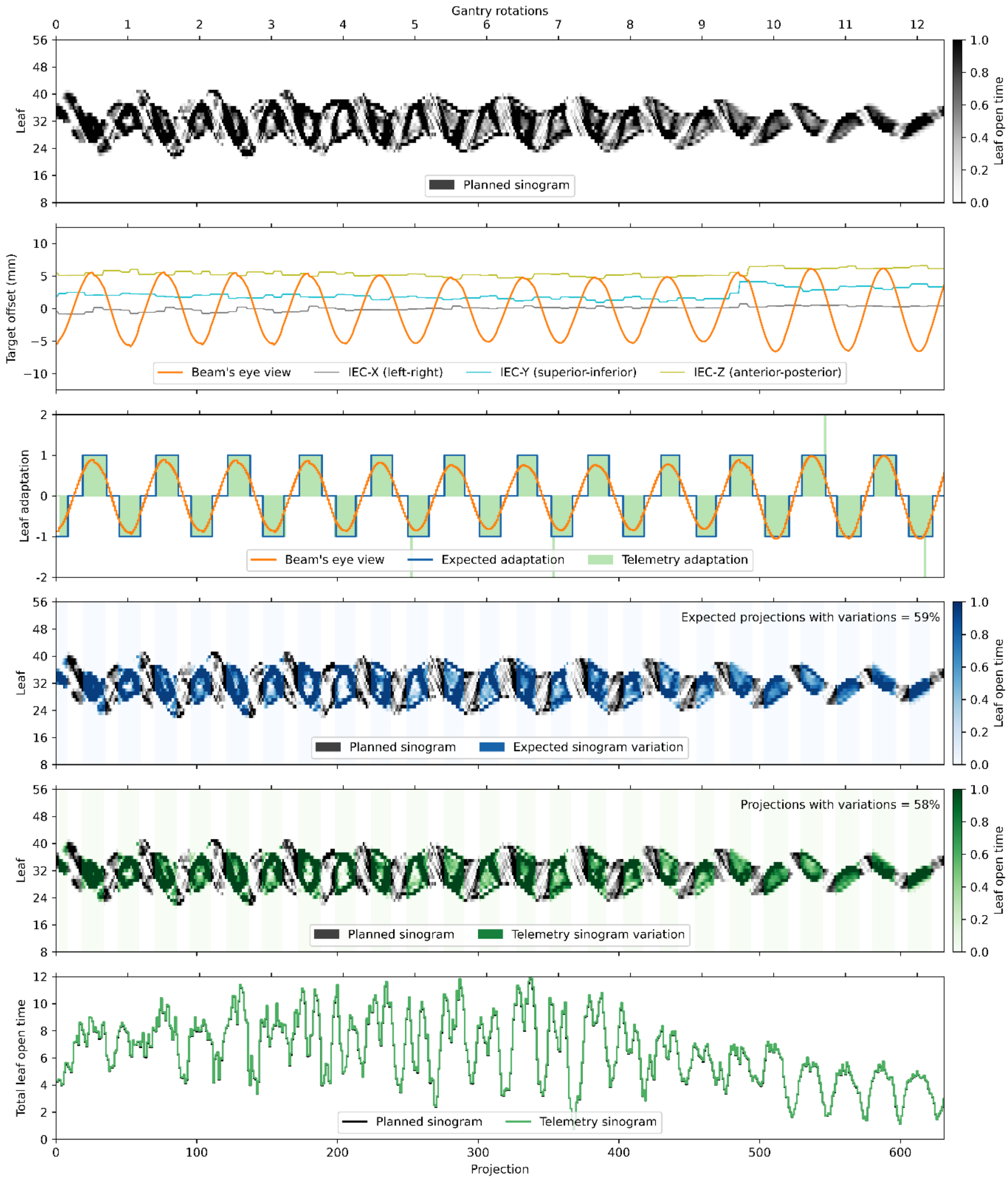

Figure 2 Example of accurate leaf behavior for prostate treatment

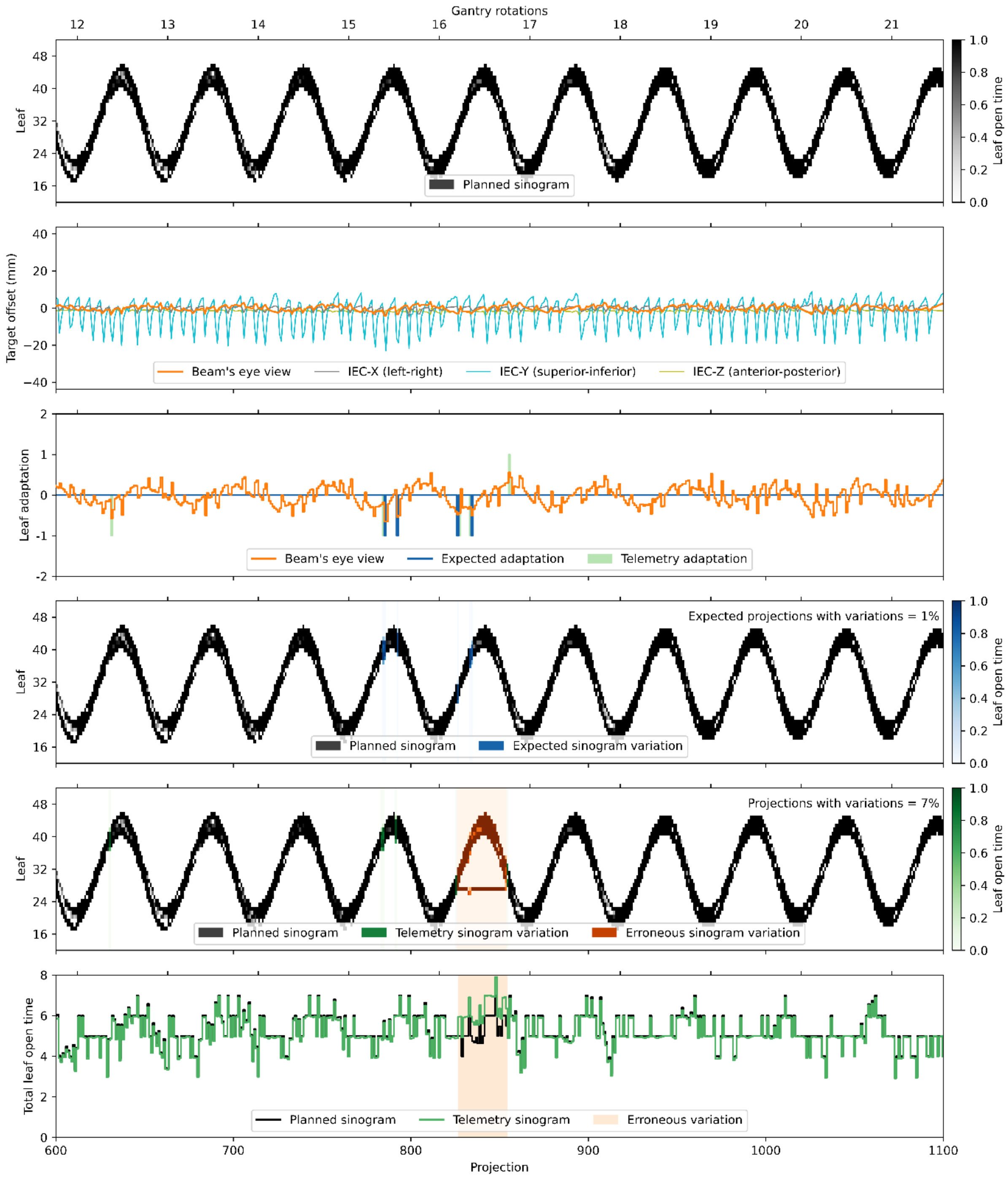

Figure 3 Example of erroneous leaf behavior for lung treatment

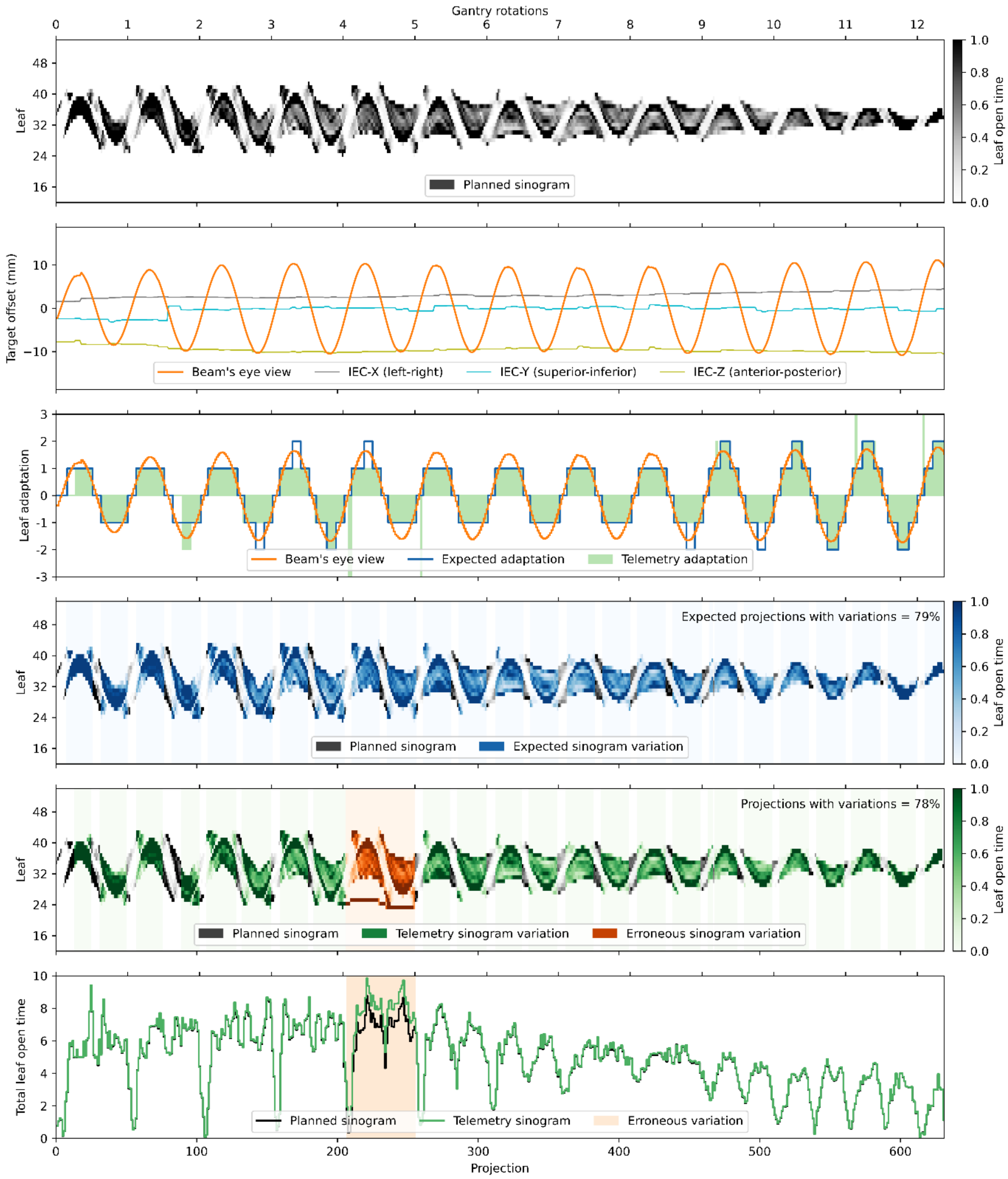

Figure 4 Example of erroneous leaf behavior for prostate treatment

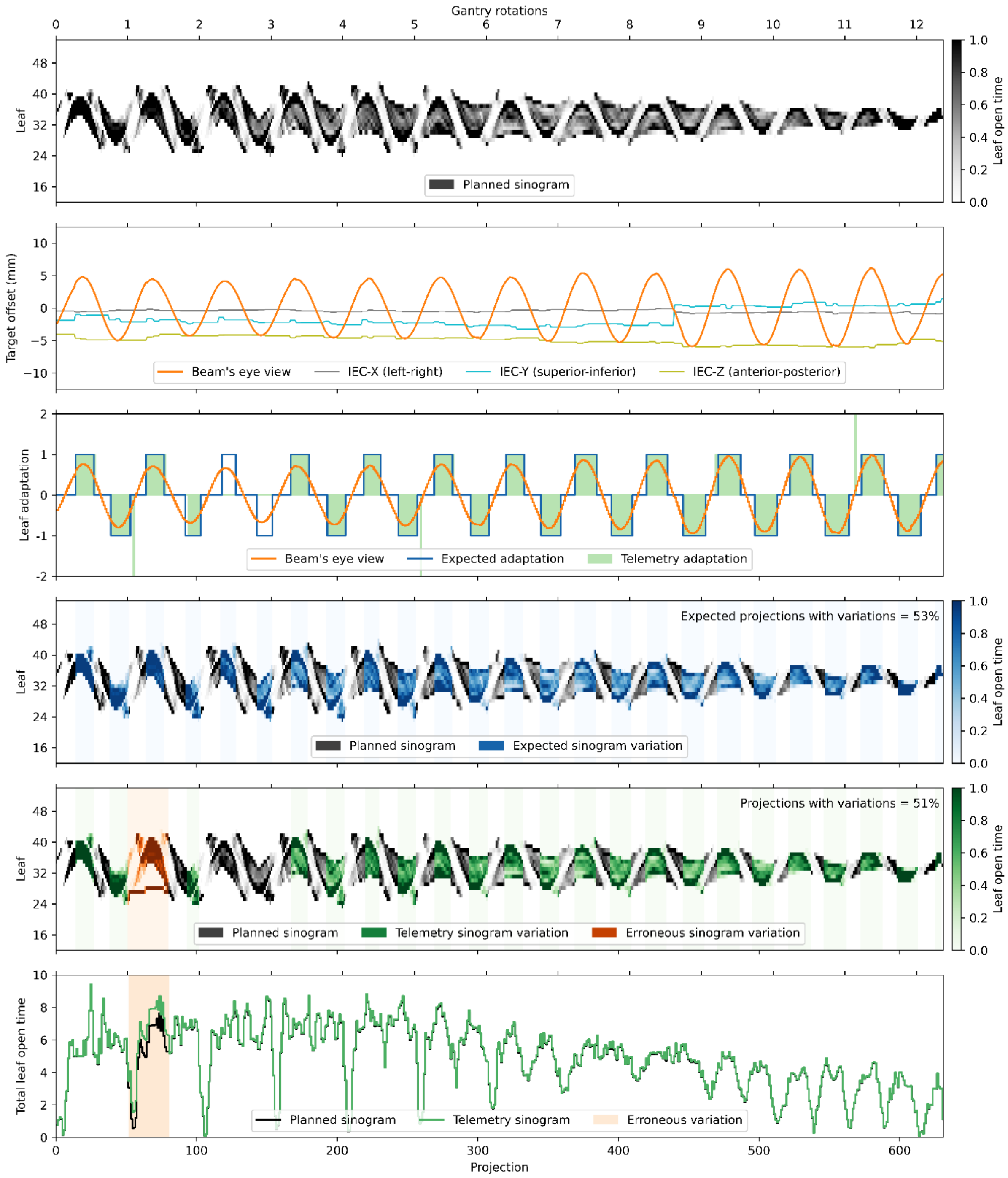

Figure 5 Example of erroneous leaf behavior for prostate treatment

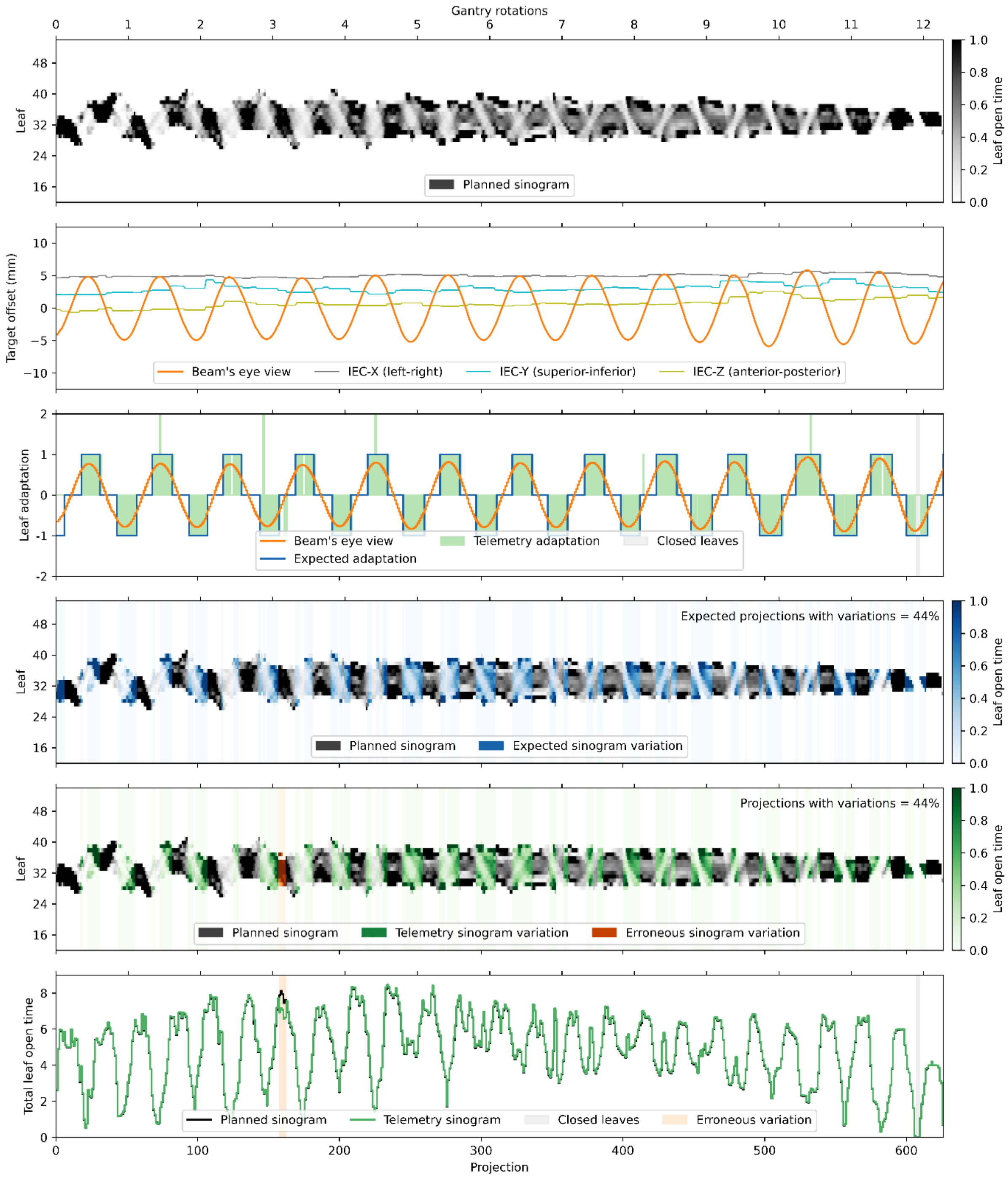

Figure 6 Example of erroneous leaf behavior for prostate treatment

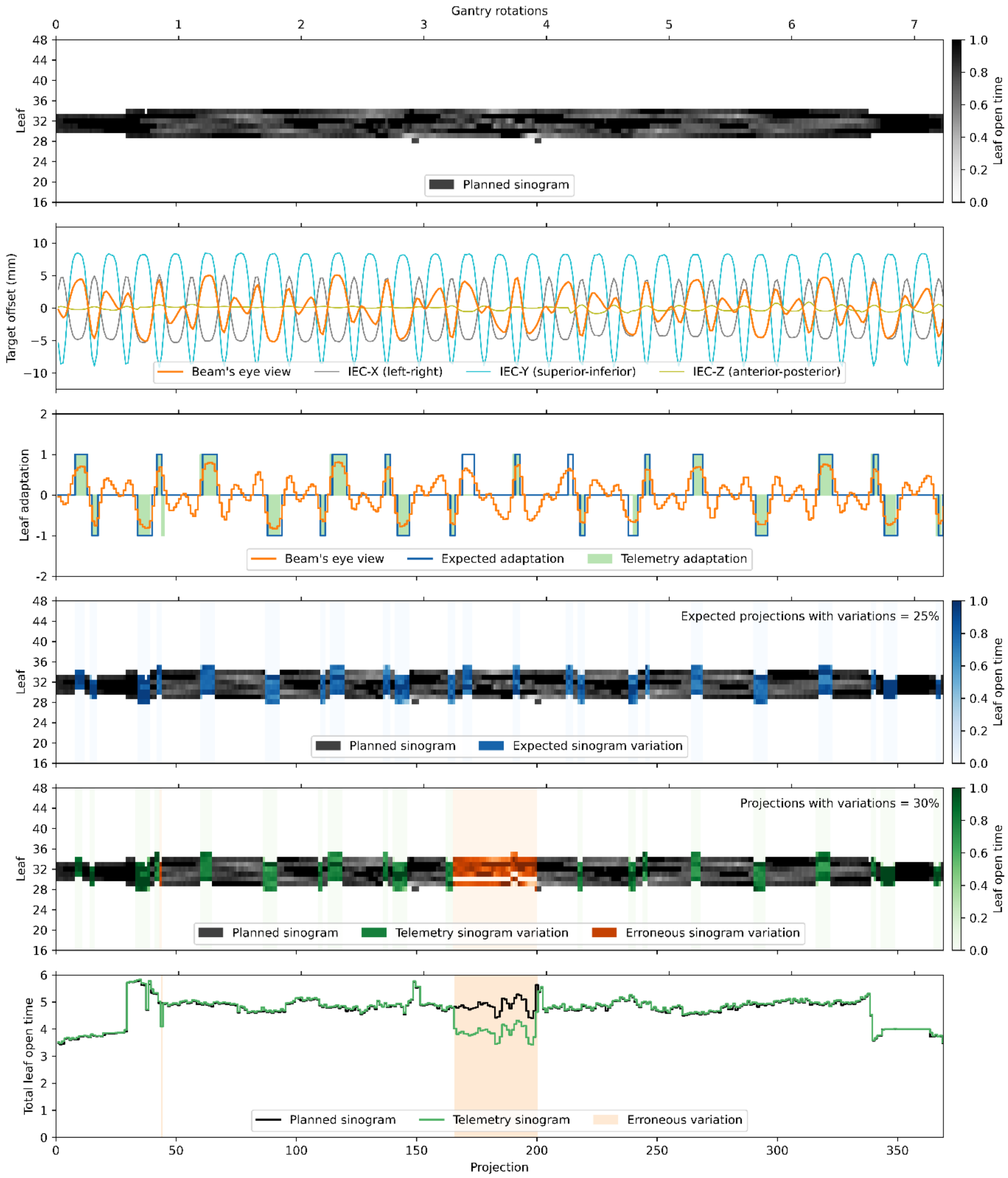

Figure 7 Example of erroneous leaf behavior for phantom treatment

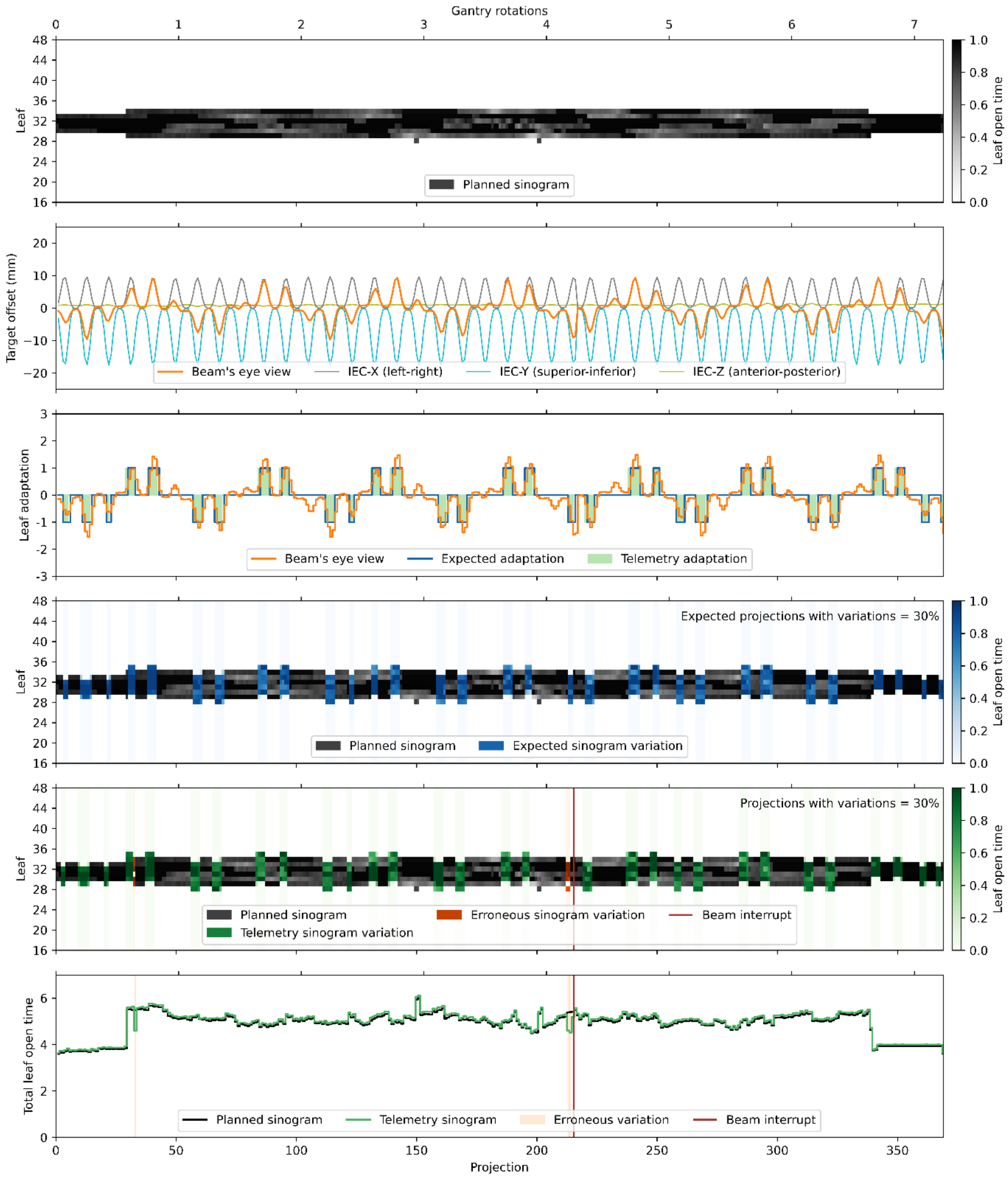

Figure 8 Example of erroneous but interrupted leaf behavior for phantom treatment
