# Supplementary material for: Retrospective log‐based quality assurance of real‐time MLC adaptation on a helical tomotherapy platform
Source: J Appl Clin Med Phys. 2026 Oct 8;27(10):e70855. doi: 10.1002/acm2.70855 (PMC13650519; doi:10.1002/acm2.70855)
Supplement: Supplementary file 3 [file ACM2-27-e70855-s003.pdf]

This Supporting Materials document provides code snippets which illustrate how files are parsed, timing data is associated with telemetry sinograms, target offset is calculated, and expected adaptation is estimated. This code has been adapted from the code available in the Git repository for illustration purposes. This has required some simplification (e.g., in the Git repository, most of these methods are encapsulated within classes).

## Table of Contents


Snippet 1: Imported modules ........ 1

Snippet 2: Reading sinograms from dplan files ........ 1

Snippet 3: Reading motion from xml files ........ 3

Snippet 4: Reading timing data from dat files ........ 4

Snippet 5: Apply timing to telemetry sinogram ........ 5

Snippet 6: Upsampling of sinogram ........ 6

Snippet 7: Calculate estimated motion adaptation ........ 8

Snippet 8: Downsampling of sinogram ........ 10


# Snippet 1: Imported modules

The following snippet describes modules that must be imported for use in subsequent snippets.

```
import binascii
import os
import numpy as np
import numpy.typing as npt
import polars as pl
```

# Snippet 2: Reading sinograms from dplan files

The following snippet describes how plan and telemetry sinogram data is parsed from .dplan files, contained in Patient Data Extractor archives.

```
def read_dplan(path: str | os.PathLike) -> pl.DataFrame:
    """Reads planned or telemetry sinogram from a .dplan file.

    Parameters
    ----------
    path : str | os.PathLike
        Path to the .dplan file.

    Returns
    -------
    pl.DataFrame
        The dplan file is returned as a Polars dataframe.

```

```
    Notes
    -----
    The function converts the binary-to-ASCII (hexadecimal) encoded data to a
    sinogram. This depends on the presence of fragment counts to determine how
    many projections exist for each leaf channel. For each leaf channel, the
    number of projections with open leaves is calculated from paired '>f8' tau
    values.
    """
    # Read dplan file
    with open(path) as text_file:
        dplan_text = text_file.read()
    dplan_text = dplan_text.split("\n")
    seconds_per_tau = float(dplan_text[4].replace("fragment.secondsPerTau=", ""))
    start_tau = int(dplan_text[5].replace("fragment.startTau=", ""))
    end_tau = int(dplan_text[6].replace("fragment.endTau=", ""))
    tau_diff = end_tau - start_tau
    counts = [
        int(i) for i in dplan_text[7].replace("fragment.counts=", "").split(" ")
    ]
    dt = np.dtype(">f8")
    bin = np.frombuffer(binascii.unhexlify("".join(dplan_text[9:-12])), dtype=dt)
    data = np.zeros((tau_diff, 64))
    offset = 0
    # Calculate leaf open times
    for index, count in enumerate(counts[:-7]):
        if count > 0:
        leaf_open_tau = bin[offset : offset + count]
        leaf_open_tau = leaf_open_tau - start_tau
        leaf_open_tau = leaf_open_tau.reshape(int(len(leaf_open_tau) / 2), 2)
        for tau in leaf_open_tau:
            data[int(tau[0]) - 1, index] = tau[1] - tau[0]
        offset = offset + count
    tau = np.arange(start_tau, end_tau, 1)
    # Calculate gantry angle, assuming projection 1 is centred on gantry 0
    gantry_angles = ((tau % 51) * (360 / 51) - (180 / 51)) % 360
    # Calculate nominal delivery times
    times = np.array(range(len(tau))) * seconds_per_tau
    # Return data if no telemetry timing available
    df = pl.DataFrame(
        {
            "projection": tau - tau[0] + 1,
            "tau": tau,
            "time": times,
            "gantry_angle": gantry_angles,
            "channel_values": data,
        }
    )
        return df
```

# Snippet 3: Reading motion from xml files

The following snippet describes how target offset data is parsed from .xml files, contained in Patient Data Extractor archives.

```
def read_xml(path: str | os.PathLike) -> pl.DataFrame:
    """Extracts motion data from *motionData.xml.

    Parameters
    ----------
    path : str | os.PathLike
        Path to XML file containing motion data.

    Returns
    -------
    pl.DataFrame
        The Radixact Synchrony motion data as a Polars dataframe.
    """
    # Read XML root
    tree = et.parse(path)
    root = tree.getroot()
    dfs = []
    # Iterate over available motion data
    for delivery_segment in root.iter("DeliverySegment"):
        for radiation_results in delivery_segment.iter("RadiationResults"):
        for model_data in radiation_results.iter("ModelData"):
            data = []
            for datapoint in model_data.iter("DataPoint"):
            data.append(
                {
                    "segment_index": int(delivery_segment[0].text),
                    "result_index": int(radiation_results[0].text),
                    "potential_diff_tolerance": float(model_data[0].text),
                    "measured_diff_tolerance": float(model_data[1].text),
                    "timestamp": int(datapoint[0].text),
                    "potential_diff": float(datapoint[1].text),
                    "rigid_body": float(datapoint[2].text),
                    "target_offset_x": float(datapoint[3][0].text),
                    "target_offset_y": float(datapoint[3][1].text),
                    "target_offset_z": float(datapoint[3][2].text),
                    "measured_diff": float(datapoint[4].text),
                }
            )
            if len(data) > 0:
                df = pl.DataFrame(data)
                dfs.append(df)
    if len(dfs) == 0:
        return None
    # Create dataframe
```

```
    df = pl.concat(dfs)
    # Remove any non-existent data
    df = df.fill_nan(None)
    df_cleaned = df.select(
        [pl.col(col) for col in df.columns if not df[col].is_null().all()]
    )
    return df_cleaned
```

## Snippet 4: Reading timing data from dat files

The following snippet describes how telemetry delivery timing data is parsed from .dat files, contained in Patient Data Extractor archives.

```
def read_dat(path: str | os.PathLike) -> pl.DataFrame:
    """Reads telemetry timing from a .dat file.

    Parameters
    ----------
    path : str | os.PathLike
        Path to the .dat file.

    Returns
    -------
    pl.DataFrame
        The dat file is returned as a Polars dataframe.
    """
    with open(path) as timing_path_text_file:
        timing_text = timing_path_text_file.read()
    timing_text = timing_text.split("\n")
    timestamps = []
    taus = []
    for n in range(int(timing_text[1])):
        # check for empty record
        if len(timing_text[3 + n * 9]) > 0:
            # Combine epoch seconds and nanoseconds as nanoseconds
            timing_timestamps = np.array(
                [
                    x * 1000000 + y
                    for x, y in zip(
                        list(map(int, timing_text[3 + n * 9].split(","))),
                        list(map(int, timing_text[4 + n * 9].split(","))),
                    )
                    if x > 0
                ]
            )
            timing_start_taus = np.array(
                [
                    x
```

```
                for x in list(map(float, timing_text[5 + n * 9].split(",")))
                if x > 0
            ]
        )
        timing_end_taus = np.array(
            [
                x
                for x in list(map(float, timing_text[6 + n * 9].split(",")))
                if x > 0
            ]
        )
        timing_intervals = np.array(
            [
                x
                for x in list(map(float, timing_text[7 + n * 9].split(",")))
                if x > 0
            ]
        )
        # Create table of projection delivery epoch timestamps
        for timestamp, start_tau, end_tau, interval in zip(
            timing_timestamps,
            timing_start_taus,
            timing_end_taus,
            timing_intervals,
        ):
            timestamps.append(timestamp)
            timestamps.append(
                timestamp + int((end_tau - start_tau) * interval * 1000000)
            )
            taus.append(start_tau)
            taus.append(end_tau)
    df = pl.DataFrame({"tau": taus, "timestamp": timestamps})
    df = df.with_columns(
        pl.from_epoch("timestamp", time_unit="us").alias("datetime")
    )
    return df
```

## Snippet 5: Apply timing to telemetry sinogram

The following snippet describes how telemetry delivery timing data is applied to telemetry sinograms, such that each projection has a timestamp that can be used to determine target offset during the projection.

```
def apply_timing(sinogram: pl.DataFrame, timing: pl.DataFrame) -> pl.DataFrame:
    """Associate telemetry timing information with sinogram data.

    Parameters
    ----------
```

```
    sinogram : pl.DataFrame
        Telemetry sinogram data.
    timing : pl.DataFrame
        Telemetry timing data.

    Returns
    -------
    pl.DataFrame
        Sinogram with associated telemetry timing information.
    """
    # Interpolate epoch timestamps for each projection delivery.
    df = sinogram.with_columns(
        timestamp=pl.Series(
            np.interp(x=sinogram["tau"], xp=timing["tau"], fp=timing["timestamp"])
        )
    )
    df = df.with_columns(
        pl.from_epoch("timestamp", time_unit="us").alias("datetime")
    )
    return df
```

## Snippet 6: Upsampling of sinogram

The following snippet describes how a sinogram is resampled to subprojections, to allow more frequent evaluation of whether adaptation should be performed.

```
def upsample(sinogram: pl.DataFrame, frequency: int = 20) -> pl.DataFrame:
    """Upsamples sinogram to fractional projections.

    Parameters
    ----------
    frequency : int, optional
        Frequency of sampling, or number of discrete points per projection.
        Default is 20.

    Returns
    -------
    pl.DataFrame
        The sinogram upsampled to specified number of points per projection.
    """
    # Calculate new projection, tau, time and gantry angles.
    curr_tau_interval = sinogram["tau"][1] - sinogram["tau"][0]
    curr_tau_start = sinogram["tau"][0]
    curr_tau_end = sinogram["tau"][-1] + curr_tau_interval
    curr_time_interval = sinogram["time"][1] - sinogram["time"][0]
    new_tau_interval = 1 / frequency
    new_tau = np.arange(curr_tau_start, curr_tau_end, new_tau_interval)
    new_projection = [int(x) for x in (new_tau - new_tau[0] + 1)]
```

```
    new_time = (new_tau - new_tau[0]) * curr_time_interval
    new_gantry_angles = ((new_tau % 51) * (360 / 51) - (180 / 51)) % 360
    # Create new data frame for updated leaf open times.
    df = pl.DataFrame(
        {
            "projection": new_projection,
            "tau": new_tau,
            "time": new_time,
            "gantry_angle": new_gantry_angles,
         }
    )
    # Calculate new leaf open times for resampled projections
    new_leaf_open_times = []
    for upsampled_row in df.iter_rows(named=True):
        upsampled_row_leaf_open_times = []
        downsampled_row = sinogram.filter(
            pl.col("projection") == upsampled_row["projection"]
        )[0]
        upsampled_row_tau_start = upsampled_row["tau"] % 1
        upsampled_row_tau_end = (upsampled_row["tau"] + new_tau_interval) % 1
        upsampled_row_tau_interval = upsampled_row_tau_end - upsampled_row_tau_start
        # Calculate new leaf open time for leaf channel
        for downsampled_leaf_open_time in downsampled_row["channel_values"][0]:
            if downsampled_leaf_open_time == 0.0:
                upsampled_row_leaf_open_times.append(0)
            else:
                overlap_start = max(
                    0.5 - (downsampled_leaf_open_time / 2), upsampled_row_tau_start
                )
                overlap_end = min(
                    0.5 + (downsampled_leaf_open_time / 2), upsampled_row_tau_end
                )
                overlap = (
                    overlap_end - overlap_start
                    if overlap_start <= overlap_end
                    else 0
                )
                upsampled_row_leaf_open_times.append(
                    overlap / upsampled_row_tau_interval
                )
        new_leaf_open_times.append(upsampled_row_leaf_open_times)
    df = df.with_columns(
        channel_values=pl.Series(np.array(new_leaf_open_times))
    )
    return df
```

# Snippet 7: Calculate estimated motion adaptation

The following snippet describes how motion and leaf open time data is registered, how target offset in beams eye view is calculated, and how an adaptation is applied to leaf projections.

```
def apply_motion_adaptation(
    sinogram: pl.DataFrame,
    timing: pl.DataFrame,
    motion: pl.DataFrame,
    leaf_adaptation_mode: str = "threshold",
    leaf_adaptation_threshold: float = 4.0 / 6.25,
) -> pl.DataFrame:
    """Adapts sinogram to specified motion.

    Parameters
    ----------
    sinogram : pl.DataFrame
        The planned sinogram.
    timing : pl.DataFrame
        The telemetry timing.
    motion : pl.DataFrame
        The motion data to use to calculate expected adaptation.
    leaf_adaptation_threshold : float, optional
        The change in target offset relative to leaf thickness in the beam's eye
        view sufficient to trigger leaf adaptation.

    Returns
    -------
    pl.DataFrame
        The expected sinogram after adaptation to motion.
    """
    # Add timing data to sinogram.
    df = apply_timing(sinogram, timing)
    time_interval = df["timestamp"][1] - df["timestamp"][0]
    df = df.with_columns(
        ((pl.col("timestamp") + time_interval / 2) / 1000).alias(
            "adaptation_timestamp"
        )
    )
    # Interpolate target offset at each timestamp
    df = df.with_columns(
        target_offset_x=pl.Series(
            np.interp(
                x=df["adaptation_timestamp"],
                xp=motion["timestamp"],
                fp=motion["target_offset_x"],
            )
        )
    )
```

```
    df = df.with_columns(
        target_offset_z=pl.Series(
            np.interp(
                x=df["adaptation_timestamp"],
                xp=motion["timestamp"],
                fp=motion["target_offset_z"],
            )
        )
    )
    # Calculate offset in beams eye view
    gantry_interval = (df["gantry_angle"][1] - df["gantry_angle"][0]) % 360
    df = df.with_columns(
        ((pl.col("gantry_angle") + gantry_interval / 2) % 360).alias(
            "adaptation_gantry_angle"
        )
    )
    df = df.with_columns(
        target_offset_beams_eye_view=np.cos(
            np.deg2rad(df["adaptation_gantry_angle"])
        )
        * df["target_offset_x"]
        - np.sin(np.deg2rad(df["adaptation_gantry_angle"])) * df["target_offset_z"]
    )
    df = df.with_columns(float_leaf_shift=df["target_offset_beams_eye_view"] / 6.25)
    # Apply adaptation
    predicted_leaf_shift = []
    curr_shift = 0
    for row in df.iter_rows(named=True):
        if (
            abs(row["float_leaf_shift"] - curr_shift)
            >= leaf_adaptation_threshold
        ):
            new_shift = int(np.rint(row["float_leaf_shift"]))
            predicted_leaf_shift.append(new_shift)
            curr_shift = new_shift
        else:
            predicted_leaf_shift.append(curr_shift)
    df = df.with_columns(pl.Series("leaf_shift", predicted_leaf_shift))
    predicted_leaf_open_times = np.zeros_like(df["channel_values"])
    for i, s in enumerate(df["leaf_shift"]):
        if s > 0:
            predicted_leaf_open_times[i][s:] = df["channel_values"][i][:-s]
        elif s < 0:
            predicted_leaf_open_times[i][:s] = df["channel_values"][i][-s:]
        else:
            predicted_leaf_open_times[i] = df["channel_values"][i]
    df = df.with_columns(channel_values=predicted_leaf_open_times)
    return df
```

# Snippet 8: Downsampling of sinogram

The following snippet describes how a previously upsampled (and adapted) sinogram is resampled to whole projections.

```
def downsample(sinogram: pl.DataFrame) -> pl.DataFrame:
    """Downsamples sinogram with fractional projctions to whole projections.

    Parameters
    ----------
    sinogram : pl.DataFrame
        The telemetry sinogram.

    Returns
    -------
    pl.DataFrame
        The sinogram downsampled to whole projections.
    """
    # Calculate new projection, tau, time and gantry angles.
    new_projection = sinogram["projection"].unique()
    new_tau = sinogram["tau"].cast(pl.Int64).unique()
    new_time_interval = (sinogram["time"][1] - sinogram["time"][0]) / (
        sinogram["tau"][1] - sinogram["tau"][0]
    )
    new_time = (new_projection.to_numpy() - 1) * new_time_interval
    new_gantry_angles = ((new_tau % 51) * (360 / 51) - (180 / 51)) % 360
    # Create new data frame for updated leaf open times.
    df = pl.DataFrame(
        {
            "projection": new_projection,
            "tau": new_tau,
            "time": new_time,
            "gantry_angle": new_gantry_angles,
        }
    )
    # Calculate new leaf open times for downsampled projections.
    new_leaf_open_times = []
    for downsampled_row in df.iter_rows(named=True):
        curr_rows = sinogram.filter(
            pl.col("projection") == downsampled_row["projection"]
        )
        mean_leaf_open_times = np.mean(
            curr_rows["channel_values"].to_numpy(), axis=0
        )
        new_leaf_open_times.append(mean_leaf_open_times)
    df = df.with_columns(channel_values=pl.Series(np.array(new_leaf_open_times)))
    return df
```
